# Distribution and prevalence of refractive error in Iranian adult population results of the PERSIAN eye cohort study PECS

**DOI:** 10.1038/s41598-024-65328-2

**Published:** 2024-06-24

**Authors:** Fateme Alipour, Maryam Mohammadzadeh, Fatemeh Jafari, Alireza Lashay, Mehdi Yaseri, Nazgol Motamed-Gorji, Yousef Alizadeh, Mohammadreza Soleimani, Mohammad Mirzaei, Kourosh Shahraki, Samira Salimpour, Mohammad Reza Shoja, Gholamreza Khataminia, Abolfazl Tahkor, Roya Tavakoli, Mohammad hossein Somi, Fariborz Mansour-Ghanaei, Farahnaz Joukar, Alireza Ansari-Moghaddam, Nader Saki, Hassan Hashemi

**Affiliations:** 1 Translational Ophthalmology Research Center, Farabi Eye Hospital, Department of Ophthalmology, Tehran University of Medical Sciences, Ghazvin Sq., Kargar St., Tehran, 1336616351 Iran; 2 Tehran University of Medical Sciences, Tehran, Iran; 3 Digestive Disease Research Institute, Shariati Hospital, Tehran University of Medical Sciences, Tehran, Iran; 4 Eye Research Center, Guilan University of Medical Sciences, Rasht, Iran; 5 Department of Ophthalmology, Rafsanjan University of Medical Sciences, Rafsanjan, Iran; 6 Department of Ophthalmology, Nikoukari Eye Hospital, Medical School, Tabriz University of Medical Sciences, Tabriz, Iran; 7 Department of Ophthalmology Health Promotion Research Center, Zahedan University of Medical Sciences, Zahedan, Iran; 8 Geriatric Ophthalmology Research Center, Shahid Sadoughi University of Medical Sciences, Yazd, Iran; 9 Department of Ophthalmology, Jundishapur University of Medical Sciences, Ahvaz, Iran; 10 Health Promotion Research Center, Zahedan University of Medical Science, Zahedan, Iran; 11 Non-Communicable Diseases Research Center, Rafsanjan University of Medical Sciences, Rafsanjan, Iran; 12 Liver and Gastrointestinal Diseases Research Center, Tabriz University of Medical Sciences, Tabriz, Iran; 13 Gastrointestinal and Liver Diseases Research Center, Guilan University of Medical Sciences, Rasht, Iran; 14 Health Promotion Research Center, Zahedan University of Medical Sciences, Zahedan, Iran; 15 Department of Otolaryngology, Head & Neck Surgery, Ahvaz Jundishapur University of Medical Sciences, Ahvaz, Iran; 16 Noor Ophthalmology Research Center, Noor Eye Hospital, Tehran, Iran

**Keywords:** Diseases, Health care, Medical research

## Abstract

The Persian Eye Cohort Study, a population-based cross-sectional study from 2015 to 2020, examined refractive error prevalence among 48,618 Iranian adults aged 31 to 70. The study encompassed six centers in Iran, employing random cluster sampling for demographic, medical, and socioeconomic data collection through interviews. Ophthalmic exams included visual acuity, automated and manual objective refraction, subjective refraction, slit lamp, and fundus examinations. Using the spherical equivalent definition, the sample population was categorized into groups. Results indicated a mean age of 49.52 ± 9.31 and a mean refractive error of 0.26 diopters (D) ± 1.6 SD (95% CI − 0.27 to -0.24), ranging from -26.1 to + 18.5 SD. Prevalence of myopia (< −0.5D) and hyperopia (> + 0.5D) was 22.6% (95% CI 22.2–23%) and 12.5% (95% CI 12.1–12.8%), respectively. Regarding different age groups, the prevalence of hyperopia and astigmatism exhibited a steady and significant rise with increasing age (*p*-value < 0.001 for both). The prevalence of Myopia, however, showed a distinctive pattern, initially increasing in adults under 45, declining in those aged 55–64, and rising again among individuals aged 60 and older. Female gender, older age, urban residency, higher education, higher income, and Fars ethnicity were significantly related to a higher prevalence of myopia (*p*-value < 0.001 for all). Female gender (*p*-value < 0.001), aging (*p*-value < 0.001), urban residency (*p*-value = 0.029), and lower-income (*p*-value = 0.005) were significantly related to higher prevalence of hyperopia. Astigmatism (> 1D) was prevalent in 25.5% of participants (95% CI 25.1–25.9%) and correlated with male gender, aging, urban residency, illiteracy, and higher income (*p*-value < 0.001, < 0.001, < 0.001, < 0.001, 0.014, respectively). The study’s comparison with regional and international surveys highlighted the increase in myopia among those over 65 due to higher nuclear cataract rates in older adults. Myopia positively related to education, income, and urban residency, while hyperopia did not exhibit such associations.

## Introduction

Refractive error (RE) is the leading cause of avoidable visual impairment, according to VISION 2020^1^. World Health Organization (WHO) reported at least 2.2 billion people with visual impairment globally, almost half of which have preventable causes^2,3^. RE which can easily be treated with glasses, contact lenses, or surgery; if left uncorrected can become the primary cause of moderate and severe vision impairment (MSVI) and the second major cause of blindness. Among various refractive errors, myopia remains and will likely remain the most prevalent type^4^. Uncorrected refractive error (URE) has more significant potential impacts on global economics than any other avoidable eye disease^4,5^. It can cause decreased quality of life, social activity limitations, and poor education and employment, eventually leading to a national health and economic burden^6^. There is a global concern regarding the coverage and quality of eye care services for refractive errors. In 2011, the WHO aimed to eliminate visual impairment due to URE. They envisioned achieving this goal by increasing national awareness, improving diagnosis, and effectively correcting URE. World Report on Vision defined and introduced metrics for measuring the effective refractive error coverage (eREC)^7^.

The limitations for achieving this goal are the lack of adequate data on the prevalence and type of RE in different populations, inadequate qualitative data on the social impact, and the lack of the most cost-effective routes for providing preventive or corrective services^8^. Therefore, more population-based studies are needed to reveal the pattern of RE prevalence around the world.

The diversity in reported prevalence rates among different populations stems from differences in the definition of RE, age groups, socioeconomic status, genetic and ethnic background, environmental factors, and healthcare accessibility^9,10^. It is estimated that most people with MSVI reside in low and middle-income countries, particularly in East, Middle East, and South-East Asia^11,12^. Previous reports have even suggested a myopia “epidemic” in Asian countries compared to Western counterparts^13^. Genetics and the environment both play key roles in the development of myopia. Genome-wide association meta-analyses have demonstrated a high genetic correlation between Europeans and Asians in refraction. Therefore, the substantial disparities in myopia prevalence observed between countries cannot be solely attributed to genetic differences^14^. High education, near work, and urbanization are risk factors that may contribute to the increasing prevalence of myopia in the twentieth century^15^. Other environmental-associated potential factors include reduced time outdoors, less daily light exposure, and close reading distance^16^. The rising prevalence of myopia in these regions necessitates more population-based studies which leads to the development of new public health policies aimed at providing clinical services to prevent visual impairment among working adults. Moreover, many epidemiologic studies in Asia were conducted among school-aged or young adult populations, underscoring the importance of conducting epidemiological studies among adults.

Iran is one of the low-middle-income countries in Middle East Asia^17^. To date, population-based studies on ophthalmic disease epidemiology in Iran are limited to scattered studies, including Tehran^18–20^, Shahroud^21^, Mashhad^22^, Yazd^23^, and Zahedan^24^ Eye Studies. Based on a single-center approach, all these population-based studies cannot genuinely represent the ethnic diversity in Iran. These factors emphasize the importance of epidemiologic studies on the prevalence of REs in Iran. This study aimed to investigate the prevalence of RE and potential socioeconomic lifestyle risk factors affecting the development of eye disease among the Iranian population.

## Material and methods

### Study design and data collection

The PERSIAN Eye Cohort Study (PECS) is the ophthalmic component of the PERSIAN Cohort (Prospective Epidemiological Research Studies of the Iranian Adults), which was designed at the Farabi Eye Hospital, Tehran University of Medical Sciences. By selecting six cohort sites representing major Iranian ethnicities and different climates (Hoveizeh, Rafsanjan, Khameneh, Some’e Sara, Yazd, and Zahedan), we used a cluster sampling procedure to recruit Iranian citizens over 30 years from 2015 to 2020. Out of the entire PERSIAN Cohort study sample, 65,580 individuals enrolled in the PECS. The written manuscript describing its methodology in detail is accepted for publication in the Archives of Iranian Medicine(AIM)^25^. Written informed consent was collected from all participants. The central committee of PECS which is located at Farabi Eye Hospital, affiliated with the Tehran University of Medical Sciences, was responsible for the design and supervision of the whole study. This committee also defined and provided the same necessary instruments for all the selected centers. All optometrists were trained and assessed regularly by the central committee.

This committee and the Tehran University of Medical Sciences also provided ethical approval (approval ID: IR.TUMS.DDRI.REC.1396.1) for this study and the study was performed in accordance with the Declaration of Helsinki.

### Eye examination

Optometry examination, the first and main step of the whole study, was performed by trained optometrists. At first, optometrists completed the interview questionnaire based on the self-reports then gathered data was recorded on the online web-based checklist. The obtained information included reports of diabetes history, past ophthalmic examination, past ocular surgeries, dry eye symptoms, history of wearing glasses or contact lenses, family history of glaucoma, retinitis pigmentosa, keratoconus, and retinal detachment. The recorded data included uncorrected visual acuity (UCVA), best-corrected visual acuity (BCVA), objective refraction with the auto refractometer, subjective refraction, and presence or absence of relative afferent pupillary defect (RAPD). RAPD is affected at times of unilateral or asymmetrical disease of the retina or optic nerve. Visual acuity (VA) was measured using auto Snellen chart projectors at the standard distance. Participants were asked to remove their glasses or contact lenses for UCVA measurement. Details of assessing visual acuity are mentioned in the protocol paper^25^. Objective refraction was assessed using the autorefractor for all patients. In addition, the glass parameters were measured by a lensometer. Optometrists used the slit lamp to examine the corneal opacity and eyelid lesions and to measure the intraocular pressure(IOP) with a Goldman tonometer. Additionally, the presence of strabismus was assessed with the cover-uncover test. Moreover, the optometrists took two dilated slit lamps and fundus photographs from all participants. Participants who fulfilled at least one of the criteria mentioned below were referred to the ophthalmologist for further examination: "1. Positive diabetes history, 2. Positive family history of glaucoma, 3. IOP > 20 mmHg, 4. Positive RAPD, 5. BCVA < 20/25 or 0.8 decimal on Snellen chart, 6. Documented/ suspicious strabismus, 7. Suspicious keratoconus, based on positive scissor motion sign, 8. Present eyelid abnormalities, 9. Moderate to severe dry eye symptoms, 10. Poor red reflex, 11. Any other suspicious findings."

In the second step, trained ophthalmologists examined the referred participants with the slit lamp in each center. The ophthalmologic examination included evaluating eyelids, lacrimal system, and extraocular muscles, assessing conjunctiva, cornea, anterior chamber, red reflex, and RAPD using the slit lamp.

### Definition of refractive error(REs)

To assess the refraction status, we evaluated the subjective refraction of both eyes in all subjects. Due to the high correlation in subjective refraction between the right and left eyes (Pearson’s correlation: r = 0.82 *p*-values < 0.005) in the whole population, we analyzed variables based on the right eye (OD) reports except for the anisometropia which involved both eyes in the analysis. Refractive error was defined based on the spherical equivalent (SE = sphere power + half of the cylinder power). We considered eyes with − 0.5 ≤ SE ≤ 0.5 diopter (D) as emmetropes and categorized myopic eyes as SE < -0.5D and hyperopia as SE >  + 0.5D. Moreover, astigmatism was defined as the cylindrical error > 1D. We further classified astigmatism into the following groups: with the rule (WTR) as 0 ± 19°, against the rule (ATR) as 90 ± 19°, oblique as 20–70°, and 110–160°. Myopia was classified as low myopia with − 3 ≤ SE < − 0.5D, moderate − 6 < SE < − 3D, and high myopia SE ≤ − 6D. On the other hand, hyperopia was classified as low manifest hyperopia with 1 < SE < 3D and moderate to high manifest hyperopia with SE ≥ 3D. Moreover, anisometropia was reported when there was a > 1.5D difference between both eyes. We further classified anisometropia into two categories: anisometropia with the same direction (both eyes with either myopia or hyperopia) and antimetropia (one eye myopic and the other hyperopic).

According to the highest education level, participants were categorized into four different groups including illiterate (lack of education), low (one to five years of education), High School diploma, and higher education (tertiary education/university degree). Based on their residence for the last nine months, participants were divided into urban and rural areas. Criteria for rural and urban areas have been explained in the Persian cohort protocol paper^26^. Wealth score index (WSI) was estimated by multiple correspondence analysis (MCA) which is explained in the Persian Cohort Protocol^27^.

### Statistical analysis

We used the mean, standard deviation (SD), median, range, frequency, and percentage to present data. The estimates have been presented with their related 95% confidence interval (CI). To evaluate the effect of selected sociodemographic variables on the refractive errors, considering the cluster sampling and probable design effect, we used simple and multivariable logistic regression on the multilevel analysis (Measurements on eyes as primary level and centers as second levels).

Odds ratios (ORs) with their 95% CIs are reported. All statistical analyses were performed by Stata (StataCorp. 2021. Stata Statistical Software: Release 17. College Station, TX: StataCorp LLC). A *P*-value less than 0.05 was considered statistically significant.

## Results

### Demographic characteristics

The optometric data were available from 48,618 subjects out of Out of 65,580 individuals (Response rate:74%). The mean age was 49.52 ± 9.31SD in the total sample population and 50.1 ± 9.2 SD among patients with optometry evaluation. This sample consisted of 55.5% women (26,996 subjects) and 44.5% men (21602subjects), with no significant difference, compared to PERSIAN Cohort data (55.43% female and 44.57% male)^28^. Table 1 summarizes the demographic characteristics of the entire sample population.

**Table 1:** Demographic characteristics of the population.

Gender		Male N(%)	Female N (%)	Total N (%)
		21,602 (44.5)	26,996 (55.5)	48,598
Age groups	≤ 44	6770 (40.9)	9802 (59.1)	16,572 (34.1)
	45–54	7173 (43.6)	9289 (56.4)	16,462 (33.9)
	55–64	6048 (48.4)	6455 (51.6)	12,503 (25.7)
	≥ 65	1610 (52.6)	1450 (47.4)	3060 (6.3)
Education	Illiterate	1957 (24.4)	6079 (75.6)	8036 (16.5)
	Low	5572 (36.1)	9854 (63.9)	15,426 (31.8)
	Diploma	10,540 (53.6)	9128 (46.4)	19,668 (40.5)
	Higher Education	3525 (64.7)	1927 (35.3)	5452 (11.2)
Current Residence	Urban	16,604 (44.3)	20,885 (55.7)	37,489 (77.1)
	rural	4998 (45)	6111 (55)	11,109 (22.9)
WSI	Low	5889 (36.4)	10,270 (63.6)	16,159 (33.3)
	Moderate	7007 (46.2)	8163 (53.8)	15,170 (31.2)
	High	8691 (50.4)	8546 (49.6)	17,237 (35.5)
Ethnicity	Fars	7784 (46.3)	9016 (53.7)	16,800 (34.6)
	Azari	5450 (44.8)	6719 (55.2)	12,169 (25)
	Balouch	999 (34.5)	1895 (65.5)	2894 (6)
	Arab	1485 (41.7)	2078 (58.3)	3563 (7.3)
	Zaboli	2107 (41.6)	2956 (58.4)	5063 (10.4)
	Guilak	3271 (47)	3682 (53)	6953 (14.3)
	Others	506 (43.8)	650 (56.2)	1156 (2.4)
Centers	Zahedan	3934 (39.1)	6128 (60.9)	10,062 (20.7)
	Ahwaz	1445 (41.3)	2057 (58.7)	3502 (7.2)
	Rafsanjan	3973 (46.5)	4578 (53.5)	8551 (17.6)
	Gilan	3656 (46.7)	4167 (53.3)	7823 (16.1)
	Tabriz	5384 (44.9)	6599 (55.1)	11,983 (24.7)
	Yazd	3210 (48.1)	3467 (51.9)	6677 (13.7)

WSI, Wealth Score Index.

### Distribution and Prevalence of REs

While assessing the prevalence of refractive errors in the population, it was found that 65% were emmetropic (31,488 subjects, 95% CI 64.5–65.4%), and the remaining 35% of subjects (16,987) were ametropes. The mean value of SE in the entire population was − 0.26 D ± 1.6 SD (95% CI − 0.27 to − 0.24 D), with a range of − 26.1 to + 18.5 SD.

Myopes and hyperopes comprised 22.6% (95% CI 22.2–23%) and 12.5% (95% CI 12.1–12.8%) of the total population, respectively. As mentioned earlier we classified manifest myopia and hyperopia into distinct categories. As shown in Table2, low myopia (− 3 D ≤ SE < − 0.5D) and low hyperopia (1 D < SE < 3D) make up the most considerable proportion of the ametropic population (18.4% for low myopia and 11.3% for low hyperopia). The prevalence of astigmatism was found to be 25.5% (95% CI 25.1–25.9%), with oblique astigmatism being the most common type, representing 79.9% of cases (95% CI 79.6–80.3). Anisometropia, characterized by a > 1.5 D difference in both eyes, was present in 11% of the population. Anisometropia with the same direction was the most frequent type (69.2%). Table 2 also provides information on the mean SE in each category.

**Table 2:** The overall prevalence of different Refractive Errors(REs).

Refractive Errors			Number	%*	95% CI		Mean SE (D)	95% CI	
					Lower	Upper		Lower	Upper
Emmetropia			31,488	65	64.5	65.4	− 0.02	− 0.02	− 0.01
Myopia	Total		10,958	22.6	22.2	23	− 2.08	− 2.12	− 2.04
		Low	8924	18.4	18.1	18.8	− 1.3	− 1.37	− 1.35
		Moderate	1558	3.2	3.1	3.4	− 3.9	− 3.99	− 3.91
		High	476	1	0.9	1.1	− 9.5	− 9.81	− 9.14
Hyperopia	Total		6029	12.4	12.1	12.7	1.8	1.77	1.83
		Low	5459	11.3	11	11.5	1.52	1.51	1.53
		Moderate to High	570	1.2	1.1	1.3	4.47	4.3	4.63
Astigmatism	Total		12,362	25.5	25.1	25.9	− 0.9	− 0.94	− 0.86
		WTR	2730	5.6	5.4	5.8	− 1.4	− 1.49	− 1.3
		ATR	7002	14.4	14.1	14.8	− 0.56	− 0.61	− 0.52
		Oblique	38,743	79.9	79.6	80.3	− 0.12	− 0.14	− 0.11
Anisometropia	Total		5308	11	10.7	11.3	− 0.92	− 1	− 0.83
		Same Direction	3171	69.2	67.9	70.6	− 1.61	− 1.74	− 1.47
		Antimetropia	1409	30.8	29.4	32.1	0.18	0.1	0.27

CI, Confidence Interval, SE, Spherical Equivalent, D, Diopter, WTR, With The Rule, ATR, Against The Rule, *The percentages represent the prevalence of each subcategory among the total population.

Univariate and Multivariable multilevel logistic regression with different parameters were employed to identify the associative factors of different REs. The prevalence rates, *p*-values, and odds ratios are presented in Tables 3 and 4. The analysis revealed that, in both univariate and multivariate models, women exhibited a higher risk of myopia (unadjusted OR 1.05, 95% CI 1.004–1.09, *p* = 0.029 and adjusted OR 1.09, 95% CI 1.04–1.14 *p* < 0.001) and hyperopia (unadjusted OR 1.06, 95% CI 1.009–1.13, *p* = 0.022 and adjusted OR 1.19, 95% CI 1.12–1.27 *p* < 0.001) when compared to men. However, anisometropia and astigmatism both exhibited gender-based differences, with females displaying lower odds ratios compared to males, indicating a statistically significant variation in prevalence (adjusted for all other variables in Table 4, *p*-values < 0.001 for both).

**Table 3:** Prevalence rates of different refractive errors by gender, age, residency state, education, WSI, ethnicity, and centers.

Effective factors		Emmetropia		Myopia		Hyperopia	
		%	95%CI (lower–upper)	%	95%CI (lower- upper)	%	95%CI (lower- upper)
Gender	M	65.7	65–66.3	22.2	21.6–22.8	12.1	11.7–12.6
	F	64.4	63.8–65	22.9	22.4–23.4	12.7	12.3–13.1
*p*-value		0.003*		0.029*		0.022*	
Age	≤ 44	72.5	71.8–73.2	24.7	24–25.4	2.8	2.6–3.1
	45–54	66.8	66.1–67.6	21	20.4–21.7	12.1	11.6–12.6
	55–64	56.4	55.6–57.3	20.6	19.8–21.3	23	22.3–23.8
	≥ 65	48.7	47–50.5	28.1	26.5–29.7	23.2	21.7–24.7
*p*-value		< 0.001*		< 0.001*		< 0.001*	
Current Residence	Urban	63.9	63.4–64.4	24.1	23.7–24.6	14	13.4–14.7
	Rural	68.5	67.7–69.4	17.5	16.8–18.2	12	11.6–12.3
*p*-value		< 0.001*		< 0.001*		0.0138*	
Education	Illiterate	58.7	57.6–59.8	21.4	20.5–22.3	20	19.1–20.8
	Low	66.3	65.6–67.1	21.5	20.9–22.2	12.1	11.6–12.6
	Diploma	67.1	66.5–67.8	22.3	21.7–22.9	10.6	10.2–11
	Higher Education	62.6	61.3–63.8	28.6	27.4–29.8	8.9	8.1–9.6
*p*-value		< 0.001*		< 0.001*		< 0.001*	
WSI	Low	63.5	62.7–64.2	21	20.4–21.6	15.5	15–16.1
	Moderate	65.4	64.6–66.1	22.3	21.6–23	12.3	11.8–12.8
	High	66	65.3–66.7	24.3	23.7–25	9.6	9.2–10.1
*p*-value		< 0.001*		0.16		< 0.001*	
Ethnicity groups	Fars	62.8	62–63.5	27.6	26.9–28.3	9.6	9.2–10.1
	Azari	68.9	68–69.7	23.8	23.0–24.5	7.4	6.9–7.9
	Balouch	65.8	64–67.5	23.7	22.2–25.3	10.5	9.4–11.7
	Arab	65.3	63.7–66.8	18.4	17.1–19.7	16.3	15.2–17.6
	Zaboli	62.9	61.6–64.2	23.1	21.9–24.3	14	13–15
	Guilak	64.4	63.3–65.6	10.8	10.1–11.5	24.8	23.8–25.8
	Others	64.8	62–67.5	17.3	15.2–19.6	17.9	15.7–20.1
*p*-value		< 0.001*		< 0.001*		< 0.001*	
Centers	Rafsanjan	66.1	65.1–67.1	26	25.1–26.9	7.9	7.3–8.5
	Hoveizeh	65.3	63.7–66.9	18.2	16.9–19.5	16.5	15.3–17.8
	Some’e Sara	64.6	63.6–65.7	10.8	10.1–11.5	24.6	23.6–25.5
	Khameneh	68.9	68.1–69.7	24	23.3–24.8	7.1	6.6–7.6
	Zahedan	63.1	62.1–64	24.2	23.4–25.1	12.7	12.1–13.4
	Yazd	59.5	58.3–60.7	29.5	28.4–30.6	11	10.3–11.8
*p*-value		< 0.001*		< 0.001*		< 0.001*	

Effective factors		Astigmatism		Anisometropia			
		%	95%CI (lower- upper)	%	95%CI (lower- upper)		
Gender	M	26.7	26.1–27.3	12.2	11.8- 12.6		
	F	24.6	24.1–25.1	10	9.7- 10.4		
*p-*value		< 0.001*		< 0.001*			
Age	≤ 44	19.3	18.7–19.9	5.1	4.8- 5.5		
	45–54	23.2	22.6–23.9	8.3	7.9- 8.8		
	55–64	32.7	31.9–33.5	18	17.3- 18.7		
	≥ 65	41.9	40.2–43.7	28.8	27.2- 30.4		
*p*-value		< 0.001*		< 0.001*			
Current Residence	Urban	26.7	26.3–27.2	11.1	10.8- 11.4		
	Rural	21.4	20.6–22.2	10.6	10- 11.2		
*p*-value		0.038*		0.18			
Education	Illiterate	33.8	32.8–34.8	18.6	17.7- 19.4		
	Low	25.5	24.8–26.2	10.6	10- 11.2		
	Diploma	23.3	22.7–23.9	8.8	8.4- 9.2		
	Higher Education	21.3	20.3–22.5	8.8	8.5- 9		
*p*-value		< 0.001*		< 0.001*			
WSI	Low	27.4	26.7–28.1	13.5	13- 14		
	Moderate	25.6	24.9–26.3	10.7	10.2- 11.2		
	High	23.6	23–24.3	8.9	8.5- 9.4		
*p*-value		< 0.001*		< 0.001*			
Ethnicity groups	Fars	26.9	26.2–27.6	11.2	10.7–11.7		
	Azari	21.1	20.3–21.8	9.1	8.6–9.6		
	Balouch	28.8	27.2–30.5	9.2	8.2–10.3		
	Arab	36.6	35–38.2	18.9	17.6–20.2		
	Zaboli	34	32.7–35.3	13.2	12.3–14.1		
	Guilak	17.3	16.4–18.2	9.1	8.5–9.8		
	Others	21.8	19.5–24.3	9.8	8.2–11.6		
*p*-value		< 0.001*		< 0.001*			
Centers	Rafsanjan	25.1	24.2–26	10.3	9.6–10.9		
	Hoveizeh	36.7	35.1–38.3	19	17.7–20.3		
	Some’e Sara	17.6	16.7–18.4	9.1	8.5–9.8		
	Khameneh	21	20.3–21.8	9	8.5–9.6		
	Zahedan	32	31.1–32.9	12	11.4–12.6		
	Yazd	27.6	26.6–28.7	11.9	11.1–12.6		
*p*-value		< 0.001*		< 0.001*			

M, Male, F, Female, WSI, Wealth Score Index, CI, Confidence Interval, p-value of myopia was measured in comparison with the hyperopia group and wise versa.

**Table 4:** Prevalence rates of different refractive errors by gender, age, residency state, education, WSI, ethnicity, and centers.

Response			Unadjusted			Adjusted		
			OR	95% CI	*p*-value	OR	95% CI	*p*-value
				Lower–Upper			Lower–Upper	
Myopia	Gender	M (reference)	1	–		1	–	
		F	1.05		0.029*	1.09	1.04–1.14	< 0.001*
				1.004—1.09				
	Age	≤ 44 (reference)	1	–		1	–	
		45–54	0.94	0.88–0.98	0.015*	0.95	0.90–1.01	0.13
		55–64	1.06	0.99–1.1	0.063	1.08	1.02–1.15	0.008*
		≥ 65	1.67	1.5–1.8	< 0.001*	1.75	1.58–1.93	< 0.001*
	Current Residence	Urban (reference)	1	–		1	–	
		Rural	0.84	0.78–0.89	< 0.001*	0.85	0.79–0.90	< 0.001*
	Education	Illiterate (reference)	1	–		1	–	
		Low	0.87	0.8–0.92	< 0.001*	0.98	0.91–1.05	0.61
		Diploma	0.88	0.82–0.94	< 0.001*	1.02	0.94–1.10	0.6
		Higher Education	1.13	1.03–1.22	0.004*	1.3	1.2–1.47	< 0.001*
	WSI	Low (reference)	1	–		1	–	
		Moderate	0.94	0.89–1	0.06	1.03	0.97–1.08	0.2
		High	0.98	0.92–1.03	0.44	1.11	1.05–1.17	< 0.001*
	Ethnicity groups	Fars (reference)	1	–		1	–	
		Azari	0.85	0.63–1.15	0.3	0.86	0.64–1.15	0.3
		Balouch	0.75	0.65–0.85	< 0.001*	0.76	0.66–0.87	< 0.001*
		Arab	0.9	0.60–1.34	0.61	0.92	0.62–1.37	0.7
		Zaboli	0.76	0.67–0.86	< 0.001*	0.76	0.67–0.86	< 0.001*
		Guilak	0.73	0.54–0.99	0.04*	0.75	0.55–1.01	0.06
		Others	0.75	0.61–0.92	0.007*	0.75	0.61–0.92	0.007*
Hyperopia	Gender	M (reference)	1	–		1	–	
		F	1.06	1.009–1.13	0.022*	1.19	1.12–1.27	< 0.001*
	Age	≤ 44 (reference)	1	–		1	–	
		45–54	4.6	4.14–5.11	< 0.001*	4.6	4.16–5.14	< 0.001*
		55–64	11.3	10.2–12.6	< 0.001*	11.3	10.2–12.6	< 0.001*
		≥ 65	13.9	12.2–15.8	< 0.001*	13.8	12.04–15.8	< 0.001*
	Current Residence	Urban (reference)	1	–		1	–	
		Rural	0.91	0.84–0.98	0.014*	1.08	1.02–1.16	0.008*
	Education	Illiterate (reference)	1	–		1	–	
		Low	0.54	0.50–0.59	< 0.001*	0.94	0.87–1.03	0.2
		Diploma	0.43	0.40–0.47	< 0.001*	0.98	0.89–1.08	0.8
		Higher Education	0.43	0.38–0.48	< 0.001*	1.01	0.88–1.16	0.8
	WSI	Low (reference)	1	–		1	–	
		Moderate	0.84	0.78–0.90	< 0.001*	0.96	0.89–1.03	0.3
		High	0.72	0.67–0.77	< 0.001*	0.88	0.81–0.96	0.005*
	Ethnicity groups	Fars (reference)	1	–		1	–	
		Azari	0.8	0.57–1.13	0.22	0.85	0.59–1.22	0.3
		Balouch	0.75	0.63–0.91	0.003*	0.89	0.73–1.08	0.2
		Arab	0.87	0.52–1.45	0.6	1.12	0.65–1.92	0.6
		Zaboli	1.05	0.89–1.23	0.5	1.03	0.86–1.22	0.7
		Guilak	1.1	0.83–1.47	0.7	1.21	0.90–1.6	0.2
		Others	1.05	0.82–1.34	0.7	1.26	0.97–1.64	0.07
Astigmatism	Gender	M (reference)	1	–		1	–	
		F	0.86	0.83–0.90	< 0.001*	0.86	0.82–0.90	< 0.001*
	Age	≤ 44 (reference)	1	–		1	–	
		45–54	1.27	1.21–1.34	< 0.001*	1.22	1.15–1.29	< 0.001*
		55–64	2.04	1.93–2.15	< 0.001*	1.87	1.77–1.98	< 0.001*
		≥ 65	3.04	2.8–3.3	< 0.001*	2.68	2.45–2.93	< 0.001*
	Current Residence	Urban (reference)	1	–		1	–	
		Rural	0.94	0.88–0.99	0.039*	0.88	0.83–0.93	< 0.001*
	Education	Illiterate (reference)	1	–		1	–	
		Low	0.74	0.69–0.78	< 0.001*	0.92	0.86–0.98	0.023*
		Diploma	0.64	0.61–0.68	< 0.001*	0.84	0.79–0.91	< 0.001*
		Higher Education	0.53	0.49–0.58	< 0.001*	0.69	0.63–0.76	< 0.001*
	WSI	Low (reference)	1	–		1	–	
		Moderate	0.89	0.85–0.94	< 0.001*	0.96	0.91–1.01	0.15
		High	0.79	0.75–0.83	< 0.001*	0.92	0.87–0.98	0.014*
	Ethnicity groups	Fars (reference)	1			1	–	
			-					
		Azari	0.76	0.60–0.95	0.02*	0.72	0.59–0.87	0.001*
		Balouch	0.85	0.75–0.97	0.017*	0.85	0.75–0.98	0.026*
		Arab	1.39	1.08–1.78	0.008*	1.4	1.14–1.73	0.001*
		Zaboli	1.08	0.97–1.22	0.147	1.08	0.96–1.2	0.183
		Guilak	0.6	0.49–0.74	< 0.001*	0.59	0.49–0.72	< 0.001*
		Others	0.71	0.60–0.85	< 0.001*	0.74	0.62–0.88	0.001*
Anisometropia	Gender	M (reference)	1	–		1	–	
		F	0.78	0.74–0.83	< 0.001*	0.8	0.75–0.85	< 0.001*
	Age	≤ 44 (reference)	1	–		1	–	
		45–54	1.6	1.54–1.84	< 0.001*	1.62	1.48–1.77	< 0.001*
		55–64	4.13	3.8–4.49	< 0.001*	3.72	3.41–4.06	< 0.001*
		≥ 65	7.57	6.81–8.42	< 0.001*	6.41	5.72–7.18	< 0.001*
	Current Residence	Urban (reference)	1	–		1	–	
		Rural	1.05	0.97–1.13	0.18	0.96	0.88–1.04	0.35
	Education	Illiterate (reference)	1	–		1	–	
		Low	0.56	0.52–0.60	< 0.001*	0.88	0.81–0.96	0.004*
		Diploma	0.45	0.41–0.48	< 0.001*	0.81	0.73–0.89	< 0.001*
		Higher Education	0.43	0.38–0.48	< 0.001*	0.79	0.69–0.91	0.001*
	WSI	Low (reference)	1	–		1	–	
		Moderate	0.76	0.71–0.82	< 0.001*	0.86	0.80–0.93	< 0.001*
		High	0.63	0.58–0.67	< 0.001*	0.8	0.73–0.87	< 0.001*
	Ethnicity groups	Fars (reference)	1	–		1	–	
		Azari	0.78	0.67–0.90	0.001*	0.74	0.65–0.84	< 0.001*
		Balouch	0.73	0.60–0.88	0.001*	0.73	0.62–0.87	< 0.001*
		Arab	1.75	1.48–2.08	< 0.001*	1.69	1.46–1.96	< 0.001*
		Zaboli	1.09	0.92–1.29	0.28	1.02	0.89–1.18	0.69
		Guilak	0.78	0.67–0.91	0.002*	0.75	0.65–0.87	< 0.001*
		Others	0.83	0.67–1.02	0.091	0.87	0.70–1.08	0.22

M, Male, F, Female, WSI, Wealth Score Index, CI, Confidence Interval.

**Figure 1:**
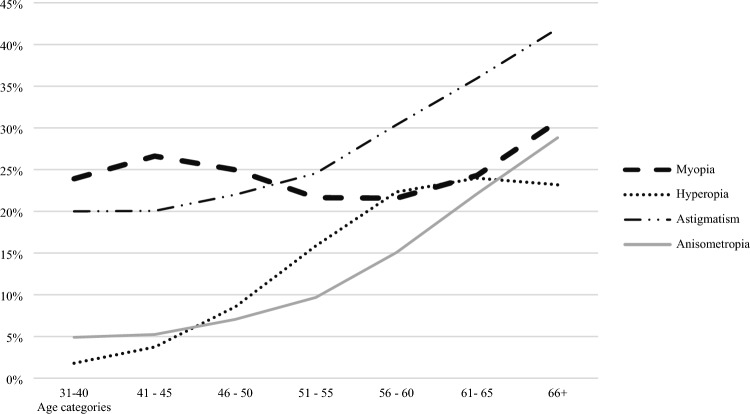
The distribution of different REs across age groups.

**Figure 2:**
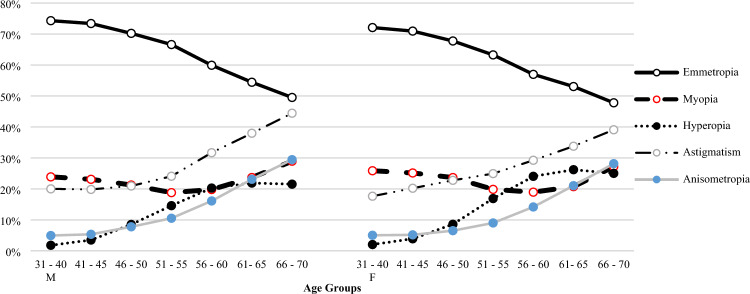
Distribution of REs among different age groups between two genders.

Regarding different age groups, it is evident that emmetropes constitute the dominant portion of the population, with a notably higher prevalence of emmetropia observed in younger individuals (< 44 years old) at 72.5%. Conversely, older age groups exhibited a higher prevalence of all refractive error types. Hyperopia and astigmatism demonstrated a gradual and significant increase in prevalence with advancing age (*p*-value < 0.001 for both as depicted in Fig. 1). However, the prevalence of myopia followed a distinctive pattern; initially rising from 23.9% among individuals aged 31–40 to 26.6% in those aged 41–45 years, as depicted in Fig. 1. This trend then reversed as age increased, reaching its nadir at 20.6% among participants aged 55–64. Subsequently, it exhibited an upward trajectory with further progression among elderly adults aged 60 and older. When compared to the reference group of individuals younger than 44 years old, myopia, hyperopia, astigmatism, and anisometropia were all significantly associated with older age groups (adjusted OR 1.75, 13.8, 2.68, and 6.41, respectively; *p*-value < 0.001 for all). Figure 2 compares the trend of emmetropia and all other refractive errors with increasing age between the two genders. Both line graphs exhibit similar trends.

Regarding the residency status, multivariable analysis revealed that individuals residing in rural regions, as opposed to urban areas, were significantly less likely to be myopic (adjusted OR 0.85, 95% CI 0.79–0.90, *p* < 0.001). A similar trend was observed for hyperopia and astigmatism (adjusted ORs 0.91, 0.88, respectively; *p*-values 0.029, and < 0.001, respectively). Individuals with higher education were more likely to be myopic or hyperopic (OR 1.3, 95% CI 1.2–1.47 and 1.01, 95% CI 0.88–1.16, respectively) compared to those who were illiterate. However, this association was not statistically significant among hyperopes (*p*-value = 0.8), despite its significance among myopics (*p*-value < 0.001). In contrast to myopia and hyperopia, astigmatic individuals were less likely to have higher educations (OR 0.69 95% CI 0.63–0.76, *p*-value < 0.001). Furthermore, an association was observed between myopia in citizens with elevated World Socioeconomic Index (WSI) (OR 1.11 95% CI 1.05–1.17, *p*-value < 0.001) while this trend was the opposite among individuals with hyperopia (OR 0.88 95% CI 0.81–0.96, *p*-value < 0.005) and astigmatism (OR 0.92 95% CI 0.87–0.98, *p*-value = 0.014). In regard to different ethnic groups, myopia exhibited a significantly higher prevalence among Fars, and logistic regression analysis revealed a significant association between myopia and Fars ethnicity when compared to Balouch (OR 0.76, 95% CI 0.66–0.87, *p*-value < 0.001) and Zaboli ethnicities (OR 0.76, 95% CI 0.67–0.86, *p*-value < 0.001). Hyperopic individuals were associated with Arab, Zaboli, and Guilak ethnic groups (OR 1.12, 1.03, 1.21, respectively), but none of them were significant.

Figure 1 shows the distribution of all refractive errors among different age groups within the entire population. Generally, the prevalence of all RE types gradually increased with increasing age. Figure 2 compares this increasing trend between two different genders. Both genders showed almost identical trends.

## Discussion

The PERSIAN Eye Cohort Study (PECS) is a population-based cross-sectional study of RE prevalence among the Iranian adult population. This report mainly provides data on the refractive status (including the prevalence of hyperopia, myopia, and astigmatism) based on age and gender. Some scattered epidemiological studies have addressed the prevalence of REs in the Iranian adult population. The previous studies had a smaller and younger sample population focused on the residents of a single city and ethnicity in Iran^19,20,22,29–33^. Reports from population-based studies in Iran are presented in Table 5. The data from selected studies are shown in Table 6 for comparison, which only contains studies on the adult population with similar age limits. Previous reports show that myopia is highly prevalent in East Asia^12,34^. The crude prevalence of myopia (< − 0.5D) in our study (22.6%) was lower than that in the Asian studies (Japan^35^, Pakistan^36^, Singapore^37,38^, and Myanmar^39^). Notably, comparable studies conducted in Beijing^40^, Bangladesh^41^, Indonesia^42^, and Taiwan^10^ align with our findings. The prevalence of myopia was found to be approximately 21.8% in the Beijing study, which involved a population-based research of 4319 Chinese individuals aged 40 years and older^40^. Similarly, a survey in Bangladesh reported a rate of 23.8% for myopia among adults over 30 years old^41^. The Indonesian study showed a myopia percentage very similar to our study, with 26.1% of myopia^42^. Moreover, the prevalence of myopia was 19.4% among Chinese older than 65 years in Taiwan^10^. The lower rates in this study might be attributed to older age limits. Comparing our results to similarly aged American, European, and Australian populations revealed similar myopia prevalence figures among black participants of Barbados^43^ and African Americans and non-Hispanic whites of Baltimore (BES)^44^. The rates among black participants over 40 years were 21.9% and 19.4% in Barbados and BES, respectively^43,44^. This disparity in the prevalence of myopia among different countries could potentially be linked to differences in ethnicity, sample selection, age ranges, examination techniques, and refractive definitions. Another explanation for this observation could be attributed to the geographical location of our country, situated in the Middle- East, at the crossroads between East and Southeast Asia and Europe. This unique position results in an ethnically diverse population, representing a blend of both regions. Therefore, it is reasonable to expect that the prevalence rates would tend to fall within the statistical range between those of the neighboring countries. Consequently, additional population-based studies are needed to evaluate geographical patterns and provide more accurate global estimates of myopia prevalence. Furthermore, one could speculate that the rates in our study may potentially be lower than the projected national prevalence. For instance, the 2008 Mashhad Eye Study, conducted in Mashhad, one of Iran’s most populous cities, indicated a myopia prevalence of 27.2% among adults aged over 54^22^. Ziaei et al.^23^ reported a myopia prevalence of 36.5% among adults over 40 in Yazd, one of the central districts of Iran. Additionally, another unpublished study from this research team in Tehran, the capital of Iran, in 2014 among individuals over ten years reported the prevalence of myopia to be 33.2%. Additionally, an earlier study in Tehran in 2004, encompassing individuals aged five years and above, reported a myopia prevalence of 21.8%^31^. The lower prevalence of myopia in our study compared to similar studies in Iran can be attributed to our sample selection, which primarily included individuals from larger age ranges and rural or less urbanized regions. This discrepancy is further underscored by reports from the United Nations, which have documented a steady rise in urbanization rates in Iran, increasing from 64.20% in 2000 to 75.94% in 2019 and projected to reach 85.82% by 2050 ^45,46^.

**Table 5:** Comparison of REs prevalence among Iranian adults.

Study	Year of Study	City	Sample size	Age	Myopia	Hyperopia	Astigmatism (cylinder power)	Anisometropia
TES (31)	2004	Tehran	4565	≥ 5	< − 0.5, 21.8%	> + 0.5,26%	≥ 0.75, 29.6%	> 1D, 6.7%
ShECS (62,63)	2009–2010	Shahroud	4864	40- 64	≤ − 0.5, 38.3%	> + 0.5, 22.1%	> 1, 24.1%	NA
Yazd Eye Study (23)	2008–2010	Yazd	2098	40–80	< − 0.5, 36.5%	> + 0.5, 20.6%	> 0.5, 53.8%	> 1D, 11.9%
Mashhad Eye Study (22)	2008	Mashhad	3132	1–90	≤ − 0.5, All the population,17.09% > 54 years, 27.2%	> + 0.5 All the population, 41.38% > 54 years, 51.6%	≥ 0.75, 25.64%	NA

**Table 6:** Comparison of REs prevalence among adults from different countries.

Study	Year of Study	Population	Sample Size	Age	Myopia	High Myopia	Hyperopia	Astigmatism (In minus cylinder)	Anisometropia (Difference SE > 1D between two eyes)
PECS	2015–2020	Iranians	48,598	> 30	< − 0.5,22.6%	< − 6, 1%	> + 1,12.4%	> 1,v25.5%	> 1.5,11%
Blue Mountains Eye Study(BMES)	1992- 1994	Australian(57)	3654	49–97	< − 0.5,15.5%	≤ − 4, 3%	> + 0.5,57%	≥ 0.75, 37% > 1.5, 13%	13%
Melbourne Visual impairment project(MVIP)	2015	Australian(49)	4744	40–80	< − 0.5,17% < − 1, 13%	< − 5, 2.1%	> + 0.5,37%	NA	NA
Barbados Eye Study (BdES)	1996–1997	Black Adults(43)	4,709	≥ 40	< − 0.5, 21.9%	NA	> + 0.5, 46.9%	NA	NA
Baltimore Eye Study(BES)	1985–1988	African Americans and non-Hispanic whites (NHWs)(44)	5036	≥ 40	< − 0.5, 19.4% (B) 28.1% (W)	< − 6, 0.0–1.4 (B) 1.3–2.5 (W)	> + 0.5, 41.0 (B) 43.9 (W)	> 0.5, 15.8–38.3 (B) 24.4–48.9 (W)	NA
Beaver Dam Eye Study (BDES)	1987–1988	NHWs (48)	4533	43–84	< − 0.5,26.2%	< − 5, 3.8%	> + 0.5,49%	NA	NA
The Los Angeles Latino Eye Study(LALES)	2005	Latinos(64)	5927	≥ 40	≤ − 1, 16.8%	≤ − 5, 3%	NA	NA	NA
Gutenberg Health Study(GHS)	2007–2012	Germany(65)	13,959	35–74	< − 0.5, 35.1%	≤ − 6,3.5%	> + 0.5, 31.8%	> 0.5, 32.3%	13.5%
Segovia study	2008	Spanish(66)	417	40–79	< − 0.5, 25.4%	< − 5,1.9%	> + 0.5, 43.6%	> 0.5, 53.5%	12.3%
The Nigerian national blindness and visual impairment survey	2008	Nigerian(47)	13,599	≥ 40	≤ − 0.5, 16.2%	< − 5, 0.7%	> + 0.5, 50.7%	< − 0.075, 58.7%	39.2%
Tajimi study	2000–2001	Japanese(35)	3021	> 40	< − 0.5, 41.8%	< − 5, 8.2%	> + 0.5,27.9%	> 0.5, 54.0%	15.1%
Indonesia Eye Study	2004–2006	Indonesia(42)	1043	≥ 21	≤ − 1, 26.1%	≤ − 6, 0.8%	≥ + 1, 9.2%	> 1, 18.5%	15.1%
The Pakistan National Blindness and Visual Impairment Survey(NBVIS)	2002–2003	Pakistan^i^(36)	14,490	≥ 30	< − 0.5, 36.5% < − 1, 31.4%	< − 5,4.6	> + 0.5, 27.1%	> 0.75, 27.1%	NA
Beijing Eye Study	2001	Chinese(40)	4319	40–90	< − 0.5,22.9%	< − 8, 1.5%	> + 0.5,20.0%	NA	NA
Tanjong Pagar survey	1996	Chinese(37)	1232	40–79	< − 0.5, 38.7%	< − 5, 9.1%	> + 0.5, 28.4%	< − 0.5, 37.8%	15.9%
Shihpai Eye Study	1999–2000	Chinese(10) in Taiwan	1361	≥ 65	< − 0.5, 19.4% < − 1, 14.5%	< − 6, 2.4%	> + 0.5,59%	< − 0.5, 74%	21.8%
Singapore Malay Eye Survey	2004–2006	Malay(38)	2974	40–80	< − 0.5,30.7%	< − 5, 3.5%	> + 0.5,27.4%	< − 0.5, 33.3%	9.9%
The Meiktila Eye Study (MES)	2005	Myanmar(39)	1863	≥ 40	< − 0.5,51% < − 1,42.7%	< − 6, 6.5%	> + 1,15%	> + 1, 30.6%	≥ 1,35.3%
National Blindness and Low Vision Survey of Bangladesh (NBLVS)	1999–2000	Bangladeshi(41)	11,624	≥ 30	< − 0.5,22.1%	≤ − 5, 1.8%	> + 0.5, 20.6%	> 0.5, 34.6%	7.5%
Andhra Pradesh Eye Disease Study (APEDS)	1996–2000	South Indians(56)	10,293	≥ 40	< − 0.5, 36.5%	< − 5, 4.5%	> + 0.5, 18.4%	< − 0.5, 37.6%	> 0.5,13.6%
Rural population of India	2001–2003	South Indian(14)	2508	≥ 40	< − 0.5, 26.99%	SE < − 5, 3.71%	> + 0.5, 18.70%	< − 0.5, 54.78%	NA
A survey of 8102 eyes	1997	Israelians(54)	8102	≥ 40	< − 1, 18.4%	NA	> + 1,24.5%	NA	NA

It is well known that a correlation exists between the prevalence of myopia and advancing age. Most studies have shown a bimodal J-shaped trend in adults, initially showing a decrease with aging and then an increase in the late 60 s^31,36–38,41,44,47–49^. In the Bangladeshi survey, myopia was significantly more common among 30 to 39 years old compared to the 40 to 49 age group^41^. Comparable findings were documented in the Beijing study, indicating a significantly higher prevalence of myopia among younger individuals^40^. This trend represents a left shift toward younger ages in the distribution of myopia in those populations. In this study, the peak of myopia was seen among individuals older than 65 years, and the second-highest prevalence of myopia was observed in participants aged 41 to 44 years. In agreement with our results, Hashemi et al.^33^ in a five-year Shahroud eye cohort study reported a similar uptrend in myopia prevalence after 60 years. This trend can be attributed to changes in lens density and the development of nuclear cataracts in older individuals, resulting in more negative SE values and a subsequent increase in myopia prevalence^33,36,41,49,50^. Furthermore, the declining trend in myopia prevalence among individuals aged 40 to 60 coincides with a concurrent significant fall in emmetropia and a rise in hyperopia prevalence (the hyperopic shift)^37,49,51^. As we did not employ cyclo-refraction for hyperopia measurement, this could be attributed to the decrease in the prevalence of facultative hyperopia in favor of manifest hyperopia due to accommodation decline with age or the subsequent development of cortical cataracts in this group^52^. In this study, the later increase in hyperopia prevalence occurred at similar ages (> 55,50–60 years) to other studies^33,43,44,48^.

There has been diversity in the relationship between the prevalence of refractive errors and gender throughout the literature. In our study, the age-adjusted prevalence showed that myopia and hyperopia were more frequent among women, which was consistent with similar previous studies among the Iranian population^31^. Female gender was associated with a higher prevalence of myopia in NHANES^53^, Beaver Dam Eye Study^48^, and among Chinese adults in Singapore^37^. However, myopia was more frequent among males in BES^43^, Israel^54^, Indian study^55^, Nigeria^47^, and Bangladesh study^41^. In the Baltimore Eye Study, gender was not associated with myopia^44^. The higher prevalence of hyperopia among females in PECS was consistent with the reports of most other studies^37,41,43,44,47,49,56^. However, there was no association between hyperopia and gender in the Beaver Dam Eye Study^48^. All these varying reports show that there has been no established relationship between gender and REs.

We found a positive relationship between myopia and higher education. This finding was consistent with other studies^37,44,49,56,57^, which are in support of the use-abuse theory and the near work effect on the prevalence of myopia^34^. Additionally, our study found a higher prevalence of myopia, hyperopia, and astigmatism among residents living in urban areas. This could be explained by higher education among urban citizens and use-abuse theory in which myopia is triggered by close-up work^34^. This finding is consistent with reports from other studies^40,43^. We noted an association between myopia and higher WSI consistent with findings in other studies that have indicated a link to higher individual or family income^37,58^.

Astigmatism (> − 1) was present in 25.5% of the survey population. The prevalence of astigmatism in our study was similar to the Meiktila Eye Study (MES) among Myanmar adults^39^ and similar studies in Iran^31^. However, the reports from different studies should be compared cautiously because of the differences in definition, methodology, and the population age. Astigmatism showed a significant increase with age, supported by other studies^37,39,40,44,57,59^. However, due to the cross-sectional design of this study, this finding may not draw direct conclusions regarding the changes in astigmatism over a lifetime. Notably, the prominent type of astigmatism observed in our study was oblique and against the rule astigmatism aligning with findings from previous population-based studies conducted among Malay adults of Singapore^38^, Bangladeshi^41^, and Indian adults^60^. In contrast, Hashemi et al.^31^ found that with the rule astigmatism was the most common type of astigmatism in the Tehran Eye Study. They reported a decreasing trend in the prevalence of with the rule and an increasing one in the prevalence of oblique and against the rule astigmatism with increasing age. Furthermore, our study revealed a higher prevalence of astigmatism among males and individuals with limited or no formal education. This association between astigmatism and lower educational attainment has also been reported in other studies^41^. The prevalence of hyperopia (> + 1D) in our study (12.4%) was lower than other similar studies in Asia and Iran. Due to our narrower inclusion criteria, which considered only individuals with SE >  + 1 D, our study reported a lower prevalence of hyperopia compared to other studies that included values >  + 0.5 D. Another reason is that we included adults over 30 in our study which makes the prevalence of hyperopia lower compared to other studies with younger sample populations^22,61^.

We found an association between hyperopia and increasing age, a trend that has also been documented in other studies^31,36,38–41,43^. Similarly, in line with previous studies^40,41^, hyperopes were more likely to have higher educations, although no significant association was found. Moreover, the observed connection between hyperopia and lower WSI in our study aligns with the lower income patterns documented in other previous studies^58^. Regarding ethnicity, we observed a link between myopia and Fars ethnicity in comparison to Balouch and Zaboli ethnicities in Iran.

This study is the most extensive epidemiologic study on the REs prevalence and associations among the Iranian adult population. Considering the importance of refractive error correction as the preventable cause of visual impairment, these studies can play a vital role in guiding the planning and implementation of refractive correction strategies on a national and global scale. Furthermore, with regard to the evolving pattern of refractive error prevalence across various geographical regions, this necessitates replicating these studies in diverse geographic areas with varying sample populations and through different time periods. This will eventually assist in calculating eREC and establishing strategies to improve accessibility and quality of eye care services^7^.

### Limitations

The PECS was originally designed as a cohort study; however, in this paper, we have exclusively presented the outcomes of the initial phase in the context of a cross-sectional study. The emergence of the COVID-19 pandemic has caused a delay in the progression of the second phase of this cohort project. Due to these circumstances, our method for monitoring the progression of refractive errors was limited to comparing different age groups. Ideally, we would have tracked the progression over time within the framework of a comprehensive cohort study. The comparison between different population-based studies is difficult because of differences in sample population, examination techniques, and definitions. One notable difference arises in cycloplegic refraction, particularly in population-based studies involving children or younger adults. Since our study focused on adults over 30 years old, this difference may introduce some bias when comparing our findings with those of studies involving younger participants.

## Data Availability

Data supporting the findings of this study, including summary statistics, are supported by The Ministry of Health and Medical Education and some aspects of them are available upon reviewer’s request. Interested parties may obtain access to the data by contacting Prof. Alireza Lashay at alirezalashay3601@gmail.com.
